# Development of One Health-based community surveillance system to study zoonotic diseases: a study protocol

**DOI:** 10.3389/fpubh.2025.1646123

**Published:** 2026-01-14

**Authors:** Debaprasad Parai, Matrujyoti Pattnaik, Shishirendu Ghosal, Chandra Prasad Sharma, Tanveer Rehman, Hari Ram Choudhary, Rachita Pradhan, Mohandoss Nagalingam, Balamurugan Vinayagamurthy, K. Vinod Kumar, S. Parthasarathy, Jyoti Ranjan Biswal, Dibyajyoti Chutia, Ipsita Pal Bhowmick, Sneha Singh, Rakesh Kumar Sahoo, Srikanta Kanungo, Krushna Chandra Sahoo, Anoop Velayudhan, Debdutta Bhattacharya, Sanghamitra Pati

**Affiliations:** 1 Department of Microbiology & One Health, ICMR-Regional Medical Research Centre, Bhubaneswar, India; 2 ICAR-National Institute of Veterinary Epidemiology and Disease Informatics, Bengaluru, India; 3 Animal Disease Research Institute, Cuttack, India; 4 North Eastern Space Application Centre (NESAC), Shillong, India; 5 ICMR-Regional Medical Research Centre, Dibrugarh, India; 6 Department of Health Research, HTA-IN, New Delhi, India; 7 Indian Council of Medical Research, New Delhi, India; 8 Academy of Scientific and Innovative Research (AcSIR), Ghaziabad, India

**Keywords:** one health, disease surveillance, zoonotic diseases, community surveillance, cohort, protocol

## Abstract

Animals are responsible for a substantial burden of infectious diseases and pose a serious threat to human health, particularly in peri-urban areas where human-animal-environment interfaces are intensified due to rapid urban expansion. This protocol aims to develop a One Health-based community surveillance system to assess the prevalence, transmission dynamics and risk factors associated with brucellosis, leptospirosis and scrub typhus in a peri-urban setting in Odisha, India. We will conduct a prospective mixed-methods cohort study over 3 years in two peri-urban blocks of Khordha district. About 1,293 households and approximately 1,200 animals will be enrolled. Data on disease prevalence, environmental risk factors, and health behaviours will be collected through structured household surveys, serological testing of human and animal populations and environmental sampling. Findings will provide longitudinal insights into zoonotic disease patterns, key transmission pathways and modifiable risk factors. Intervention packages will be formulated based on a Theory of Change (ToC) framework which will include evidence-based behaviour change communication (BCC) strategies, water, sanitation, and hygiene (WASH) interventions, animal vaccination campaigns, and public awareness initiatives. A digital surveillance platform will be developed to facilitate real-time data integration and visualization across human, animal, and environmental health sectors. The outcome will help in evidence-based recommendations for zoonotic disease prevention, enhance early detection and response strategies, and strengthen One Health collaboration. Insights from this study will contribute to national disease surveillance programs, supporting policy decisions for improved public health in low-resourced settings.

## 1 Introduction

Zoonotic diseases - those transmitted between animals and humans - pose significant threats to global health, economies, and healthcare infrastructures (1). Humans, animals, and the environment play a crucial role in maintaining and transmitting infectious zoonotic diseases (2). It is estimated that up to 75% of emerging infectious diseases and about 60% of known infectious diseases originate from zoonotic sources (3, 4). Over two billion people worldwide are affected by zoonotic diseases each year, causing nearly two million deaths and billions of dollars in economic losses (5). In low income and lower-middle income countries (LMICs), infectious diseases account for 43.7% of deaths and 15.8% of all deaths globally (4, 6). Zoonoses are responsible for some of the devastating outbreaks over last two decades (eg, SARS-CoV-2, Nipah, SARS, MERS, H7N7, Ebola, and H1N1 viruses), however, human and animal health may be more jeopardized by endemic zoonoses (7, 8).

Rising population pressures and ongoing climate change have made the interdependence of human, animal, and environmental health more evident (9). As per the 2011 census of India, around 377 million people, or 31% of the country’s total population lived in urban areas. By 2031, it is predicted that approximately 600 million Indians will reside in cities (10). Demand for livestock products has been steadily rising, primarily due to urban population expansion coupled with economic growth, and a change in dietary preferences in developing countries. This has triggered the intensification of livestock farming, often through the inappropriate management of land and natural resources that leads to environmental degradation and loss of biodiversity (11–13). These rapid changes in habitat, grazing areas and local environments also heighten the risk of zoonotic spillovers (transmission of pathogens from animals to humans), making it critical to control rising public health issues of zoonoses (14–15). To prevent such spillover events, it is essential to have an understanding of the pathogen ecology of natural hosts in addition to human–host interactions (16). One Health is a collaborative, interdisciplinary approach that recognizes the interconnection between the health of people, animals, and the environment. A One Health framework will be crucial to mitigating zoonotic threats because it provides a unifying approach to address the root causes in a more holistic manner (17–19).

The peri-urban regions are transitional zones between rural and urban, neither totally urban nor completely rural in the conventional sense, mostly a somewhat urbanised rural area serving as a buffer zone for urbanisation, migration and access to city services (20). In developing countries like India, these areas are additionally characterised by rapid urbanisation, predominant agricultural land-use, population pressure and a dynamic socio-cultural environment (21). Together, these factors are turning them into emerging hotspots for microbial exchange between humans and animals (22–23). Lack of adequate infrastructure, such as healthcare, drinking water, and waste management, makes it difficult to sustain livelihoods and puts a strain on the health of individuals and the community in most peri-urban areas (24). In India, peri-urban areas are generally regulated by several entities, including Gram Panchayats (GP: basic unit of local self-government at the village level), Urban Development and Municipal Affairs Department (state government’s town planning department), and the district administration. GPs’ ability as an administrative body and financial capacity to provide services are limited by both manpower and financial aid. The absence of community surveillance and health awareness from both human and animal health sectors limits effective disease control, leading to poor health outcomes (25).

Odisha is a state of India located in the eastern part of the country, known for its tropical climate with high humidity, longer summer, high rainfall, frequent climate-mediated cyclones and a shorter winter. Approximately 33.50% of Odisha’s geographical area is covered by forest according to the Forest Survey of India’s assessment for 2021^1^. The state consists of 30 administrative districts, among which 13 are tribal-dominated. Previous studies in Odisha have reported high prevalence rates of zoonotic diseases, especially brucellosis, scrub typhus and leptospirosis, yet a lack of One Health-based cohort studies focusing on disease transmission pathways and risk factors in peri-urban areas of the state (26–30). Recently, the National One Health Programme for Prevention and Control of Zoonoses (NOHP-PCZ) listed the prioritized zoonotic diseases severity, economic burden, pandemic potential, capacity for prevention and control, and potential for introduction or increased transmission in India. Out of those, leptospirosis can cause a significant public health burden with 11% mortality (31). Brucellosis remains one of the neglected tropical diseases despite its huge socioeconomic impact, typically on the rural population (32). Scrub typhus is a non-malarial febrile illness accounting for a 1.3 to 33.5% fatality rate in India. The actual data remains unclear due to difficulties in diagnosing and differentiating it from other febrile illnesses (26). The study aims to develop and implement a One Health approach to assess the prevalence and transmission dynamics of brucellosis, leptospirosis, and scrub typhus among human and animal populations in a peri-urban setting using a prospective cohort design and thus to evaluate the health of the community over time.

## 2 Methodology

### 2.1 Study design

We propose prospective mixed-methods cohort research combining quantitative and qualitative methods to assess brucellosis, leptospirosis, and scrub typhus in a peri-urban setting where animal husbandry and agriculture are the primary occupations. Over three years, we will follow 1,293 households, conducting one baseline survey and three follow-up assessments. The study objectives are to establish an integrated surveillance system in a peri-urban setting to

- Determine the prevalence and distribution of brucellosis, leptospirosis and scrub typhus
- Identify risk factors associated with zoonotic transmission, including close human-animal interaction, water, sanitation, and hygiene (WASH) conditions, and vaccination provision/coverage, and
- Evaluate preventive strategies, such as behavioural change interventions, and improved WASH practices.

**Figure 1:**
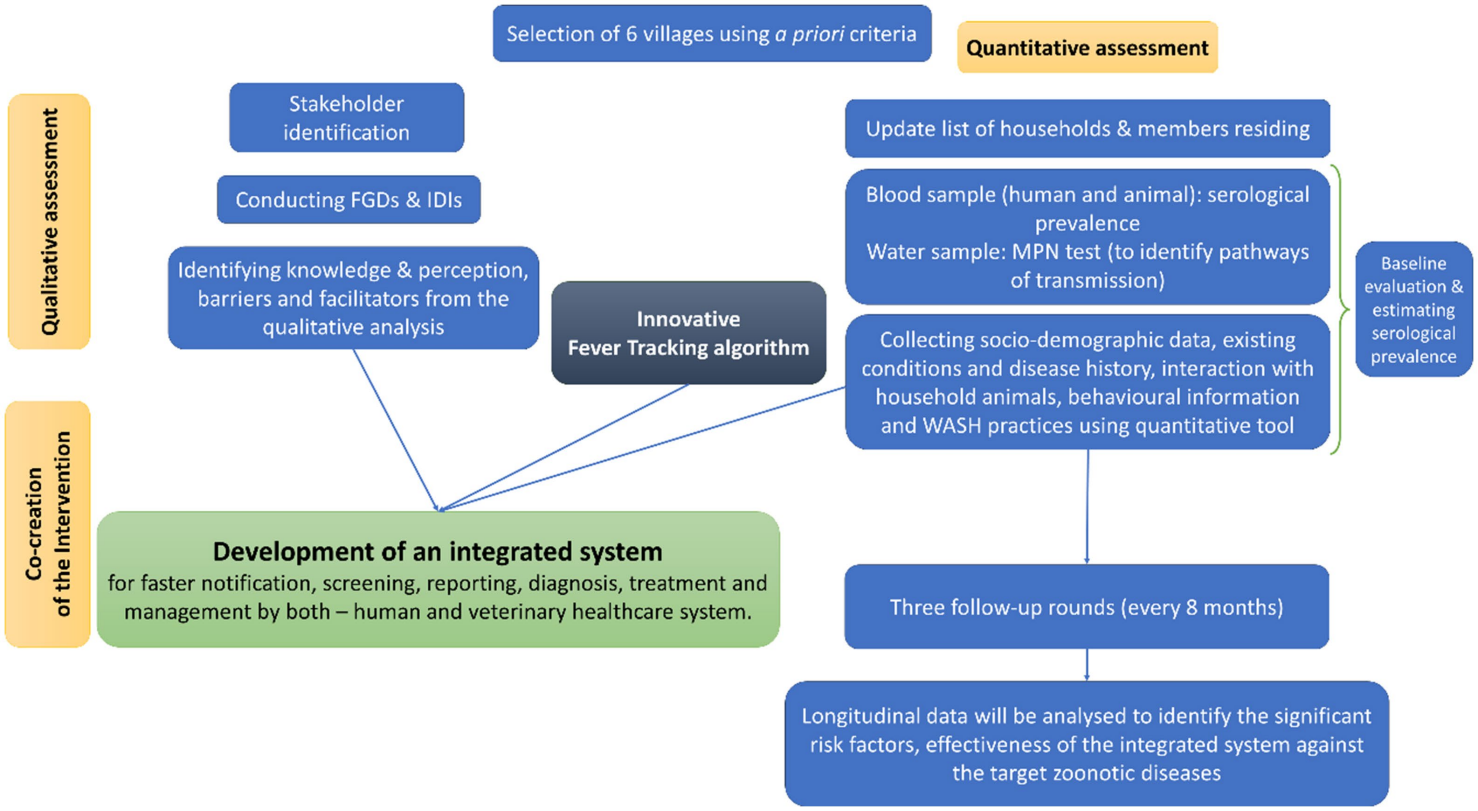
A detailed flow chart describing the study design and overview of the protocol.

Intervention packages, awareness programs, and public involvement are designed to achieve the objectives and will be discussed in detail later. A detailed flow chart describing the study design and overview is given in Figure 1. The expected outcomes are the prevalence and patterns of zoonotic diseases and their risk factors, as well as developing prevention strategies through behavioural change and WASH practices. The study’s translational value lies in linking community surveillance, laboratory diagnostics, and public health policy.

### 2.2 Study settings

**Figure 2:**
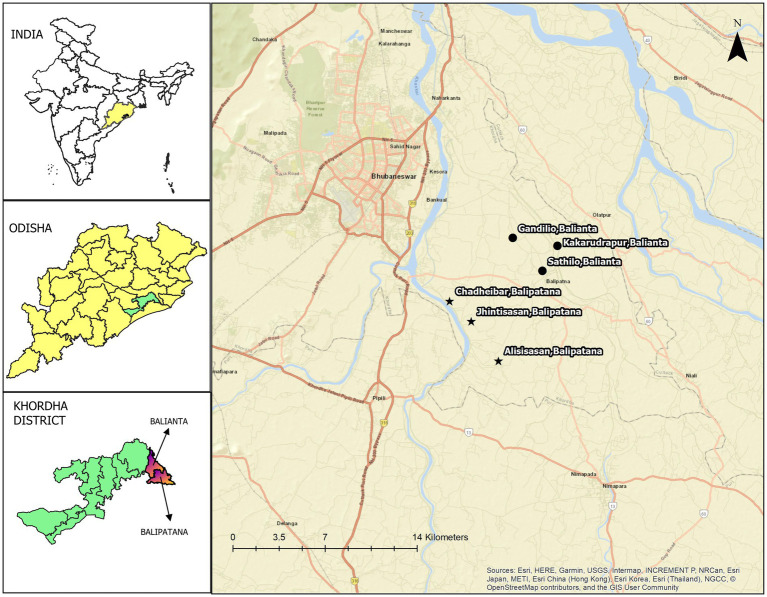
Geographical location of six peri-urban villages in Khordha district (Left) India, Odisha, and Khordha district maps created using QGIS v2.8, licensed under GNU General Public License. (Right) Map created using QGIS v3.34, licensed under GNU General Public License.

Balianta and Balipatna, two administrative blocks of the Khordha district in Odisha, were selected as the intervention sites. Their geographic location and the nearest city are presented in Figure 2. According to the latest census in 2011, the population of Balianta was 91,728 from 19,535 households. There were 46,903 males and 44,825 females. The distance between the block and the nearest city (Bhubaneswar) is 9 kilometres. Balipatna block has a total population of 132,796 people and 29,771 households. There are 67,879 males and 64,917 females among them.

**Figure 3:**
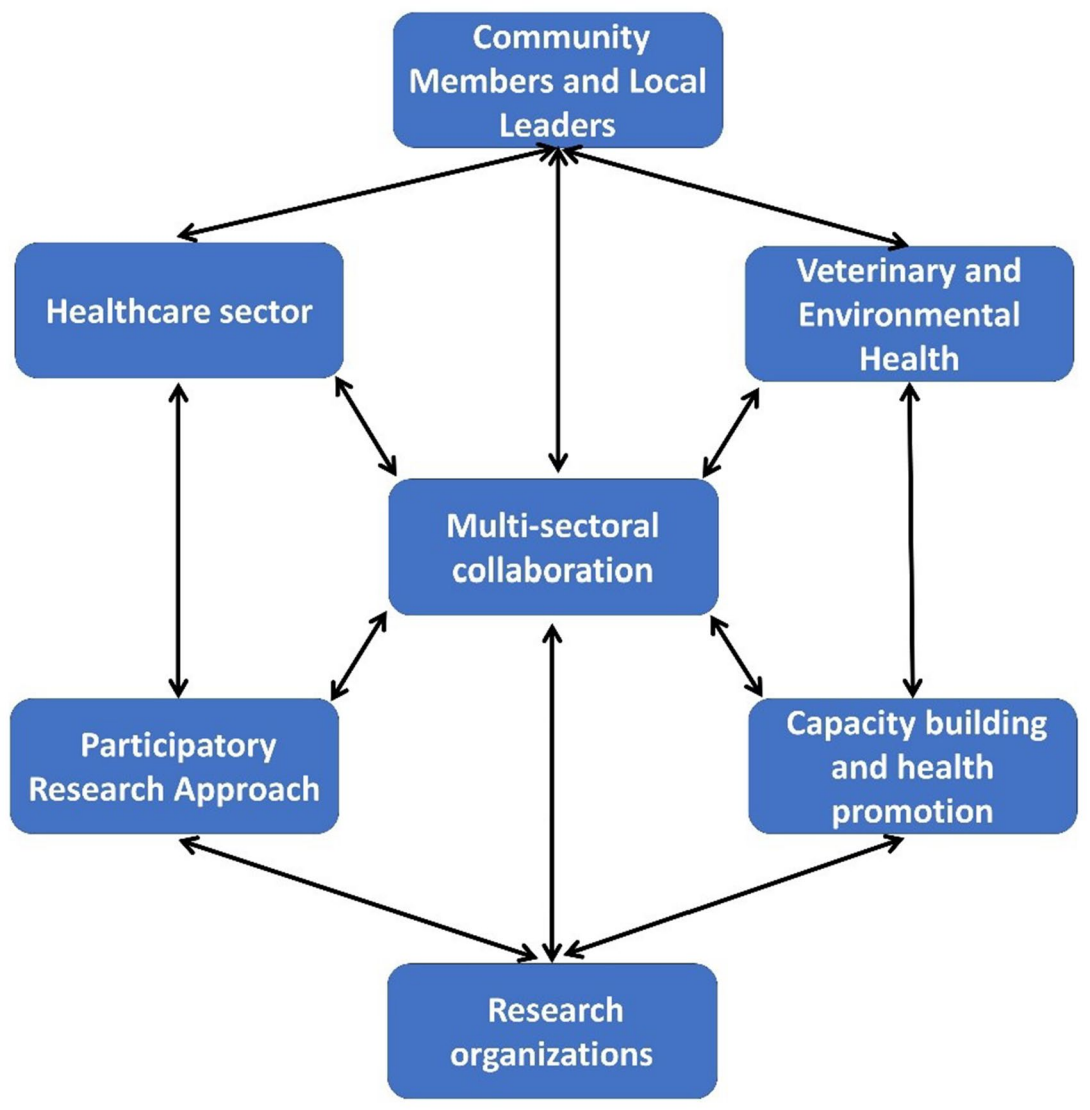
Community-based participatory approach framework for One Health cohort study in Odisha.

Three villages from each block were selected using a community-based participatory approach presented in Figure 3. The villages were chosen based on the population of various domestic livestock (Table 1) and other environmental factors such as proximity to agricultural land, poultry farms, rivers, canals, and closeness to urban centres. We selected the villages with the support of local community networks - community leaders, Panchayati Raj institutions (PRI) members, health care providers, and veterinary professionals. Free-listing and pile-sorting methodologies were used to enumerate and rank the various domestic animals in the selected villages and common animal and human diseases, including reported animal bites from the previous year. All the selected villages are located within a 12-kilometre radius of the nearest city and the highway, ensuring accessibility and a representative peri-urban setting.

**Table 1:** Variability in domestic livestock population – the primary criterion used for village selection in Balianta and Balipatna blocks in Khordha district, Odisha.

Villages	Major domestic animals
Sathilo	Cow, Goat, Poultry
Gandilo	Goat, Cow, Poultry
Kakarudrapur	Buffalo, Cow, Sheep, Poultry
Jhintisasan	Cow, Sheep, Goat, Poultry, Duck
Alisisasan	Cow, Poultry, Goat, Sheep
Chadheibar	Cow, Goat, Poultry

### 2.3 Human participants and enrolment

After liaison with the local community health centre (CHC) and the sector medical officer (doctor appointed to the primary health centre; PHC), a team of field staff will approach households along with the accredited social health activist (ASHA) to update the household line list, which will be used for calculating the final sample size and participant selection (one adult member from every household) following the inclusion and exclusion criteria given below:

Inclusion criteria

- The participant must be able and willing to comply with the study protocol, available and willing to complete all the study assessments and must have signed an informed consent form (ICF).
- Participant agrees to stay in contact with the study site for the duration of the study and provide updated and alternate contact information.
- Should be an adult resident of the Khordha district and must be residing in the study area for more than 5 years.

Exclusion criteria

- Cognitive impairment, reported by the participants or their family members.
- Children less than 18 years old will not be enrolled in the study.

### 2.4 Animal enrolment

Inclusion criteria

- Cattle, goats, and sheep from households enrolled in the study.
- Animals must belong to households participating in the study and be under the direct care of the household members.
- Animals above 6 months of age to ensure they have had sufficient exposure to environmental risk factors and potential infections.
- Clinically healthy or animals with a history of fever or reproductive disorders (abortions, stillbirths) will be included to assess disease prevalence.
- The animal must be available for baseline and follow-up sampling during the study period.
- Geographical location: the animal must reside within the peri-urban study area of Khordha district, Odisha at the time of enrolment.
- Willingness of animal owner (participant under the human study).

Exclusion criteria

- Non-owned or stray animals: only household-owned animals will be included; free-roaming or stray animals will be excluded.
- Pregnant or terminally ill or severely diseased animals: animals with severe illness, unrelated chronic conditions, or on long-term antibiotic therapy will be excluded to avoid compromising their health and adherence to ethical principles of research.
- Recently vaccinated animals (<3 months): animals vaccinated against brucellosis within the past 3 months will be excluded to prevent vaccine-induced seropositivity
- Animals already diagnosed with study diseases: if an animal has a confirmed past diagnosis of brucellosis, leptospirosis, or scrub typhus, it will be excluded from seroprevalence screening to avoid redundant testing.
- A unique identifier will be generated by adding one extra distinguishing element to the human UID, ensuring proper linkage of animal and human data for analysis. Additionally, the ear tag numbers will be noted, wherever feasible. Animal samples will be collected with the help of veterinary department, which maintains records of all animals, allowing us to cross-verify the animal data. This dual system will support reliable longitudinal tracking and data integration.

### 2.5 Sampling framework and sample size

#### 2.5.1 Human sample size

Due to the paucity of information on the prevalence of any of the target diseases in a similar setup, we conducted a pilot study where we collected blood samples from 90 participants (15 individuals, randomly selected from the line list of each village). We calculated the sample size using the formula:


$${Z_{95}^{2}}^{*}p^{*}\;(100-p)/d^{2}$$

“*Z_95_*” = Z statistic for the 95% confidence level, which is 1.96, and rounded off to 2.

“*p*” = lowest prevalence calculated among the three targeted diseases. The laboratory report from the pilot study showed the lowest prevalence of Scrub typhus IgM at 7.8% (Supplementary Table S1).

“*d*” (precision) was calculated by multiplying prevalence with the permissible error of 20% (80% power) and rounded off to 1.60.

A 20% relative precision was applied as commonly recommended in low-prevalence studies to maintain a narrow 95% CI while ensuring feasibility in terms of resource and time.

This led to a minimum sample size of 1,124. Since we will follow up the cohort for three years, another 15% of participants were added to tackle attrition, leading to a final sample size of 1,293.

#### 2.5.2 Animal sample size

During the pilot study, we estimated that 40% of the participants own animals which totals ~1,200. Blood samples will be collected from all animals with the help of the block veterinary officer (BVO), livestock inspectors and prani mitra. Blood samples will be transported in a cold chain to the designated laboratory for further processing.

### 2.6 Sampling strategies

Since we have updated the list of existing households (*n* = 1,516) and people residing in them, we will select one adult member (age ≥18 years) from each household, using the Kish-Grid method, before going for the survey. Since the number of households is quite close to the calculated sample size, we will target all the households. Using the Kish-Grid method, we shall randomly identify one adult individual from every household and inform our field team to collect quantitative information and blood samples from that person only; one research assistant will be there in the field to supervise this process. If the selected individual is not eligible to be included or does not wish to participate in the survey, the field supervisor will identify the next eligible member and so on. In this way, up to three members within one household will be approached. If participants are still not willing to participate, that household will be eliminated.

To accomplish this method, two field teams (one data entry operator and one laboratory technician in each team, along with one supervisor) will approach households along with multi-purpose health workers (MPHW – male and female) or ASHA workers. Participants who meet the eligibility criteria of the study must sign an ICF. Then, the participant will be allotted a specific Household ID by which the participant gets enrolled in the study. The quantitative information will be collected from the participants regarding socio-demographic characteristics, relevant hygiene practices, exposure history with animals and fever with treatment history. After collecting data from the participants, whole blood will be collected from them.

### 2.7 Survey tool

The Survey tool will cover identifiers and socio-demographic characteristics of the participants, their existing disease profile (especially non-communicable diseases) and previous symptoms, history of hospital visits and hospitalization, food habits and methods of preservation, household animal handling and interaction with animals. Another quantitative tool covers current water, hygiene, and sanitation practices – those could act as preventive measures. To ensure the cultural relevance and clarity of the tool, a pilot test will be conducted in two non-study sites, involving 20 participants from each location, and modifications will be made based on the feedback obtained. Trained field investigators will conduct the face-to-face interviews in the local language, utilising electronic devices for real-time data entry.

### 2.8 Follow-up strategy

All the human samples will be followed up four times, including one baseline survey and three follow-up assessments over three years. At the same time, all eligible animals will be tested at baseline. From the second round onwards, animals will be resampled under the following conditions: (a) Households where the owner is found seropositive (continued testing for disease transmission dynamics). (b) A random subset of animals from seronegative households will be monitored for potential new infections.

### 2.9 Serological investigations

All the collected human blood samples will be sent through a cold chain to the Indian Council of Medical Research – Regional Medical Research Centre (ICMR-RMRC), Bhubaneswar for further laboratory testing. Blood vials will be left undisturbed at room temperature for 30 min to allow clot formation. Then, it will be centrifuged at 2000 rpm for 5 min in a centrifuge instrument (Rotanta 460, Hettich) for serum separation. Enzyme-linked immunosorbent assay (ELISA) will be performed to test serum IgG and IgM antibodies against human brucellosis, leptospirosis, and scrub typhus infection using commercial kits (Supplementary Table S2). The final reading (optical density) will be measured in a 96-well plate reader platform (Lisascan EM, Erba Lachema) and analysed as per the manufacturer’s instructions. Internal quality assurance of the laboratory tests will be performed, which includes a daily run of quality control, periodic blind re-testing of processed and fresh samples, and weekly maintenance of the instruments. External quality control will be corroborated by a nationally accredited laboratory at ICAR-NIVEDI, Bengaluru.

Animal blood samples will be collected with the help of trained veterinarians, and serum will be separated at ADRI, Cuttack. Serum samples will then be sent to ICAR-NIVEDI for further laboratory investigations of brucellosis and leptospirosis. This laboratory is a WOAH-designated reference laboratory for leptospirosis, which participates in an annual proficiency test to ensure the quality assurance of employed serovars. Confirmation of brucellosis will be performed using the Rose Bengal plate test and ELISA. The microscopic agglutination test (MAT) will be performed for leptospirosis utilizing around 20 live serovars representing 17 serogroups, with results interpreted at the serogroup level. All samples will be screened at a 1:100 dilution, and MAT titers will be determined following WOAH guidelines (33). All serum samples will be stored at −20 °C for subsequent analysis.

### 2.10 Environmental sample testing

We will conduct geospatial mapping of the water bodies having a possibility of human-animal interactions (direct or indirect) using a GPS-enabled tablet in the six study villages. To capture the seasonal variation, the samples will be collected at six-month intervals from multiple sources, including ponds, community wells, and other water bodies with frequent human or animal contact. The quality of water from a subset will be assessed by evaluating the presence of indicator organisms by the most probable number (MPN) method and culturing on endo and eosin methylene blue agar.

### 2.11 Intervention package

**Figure 4:**
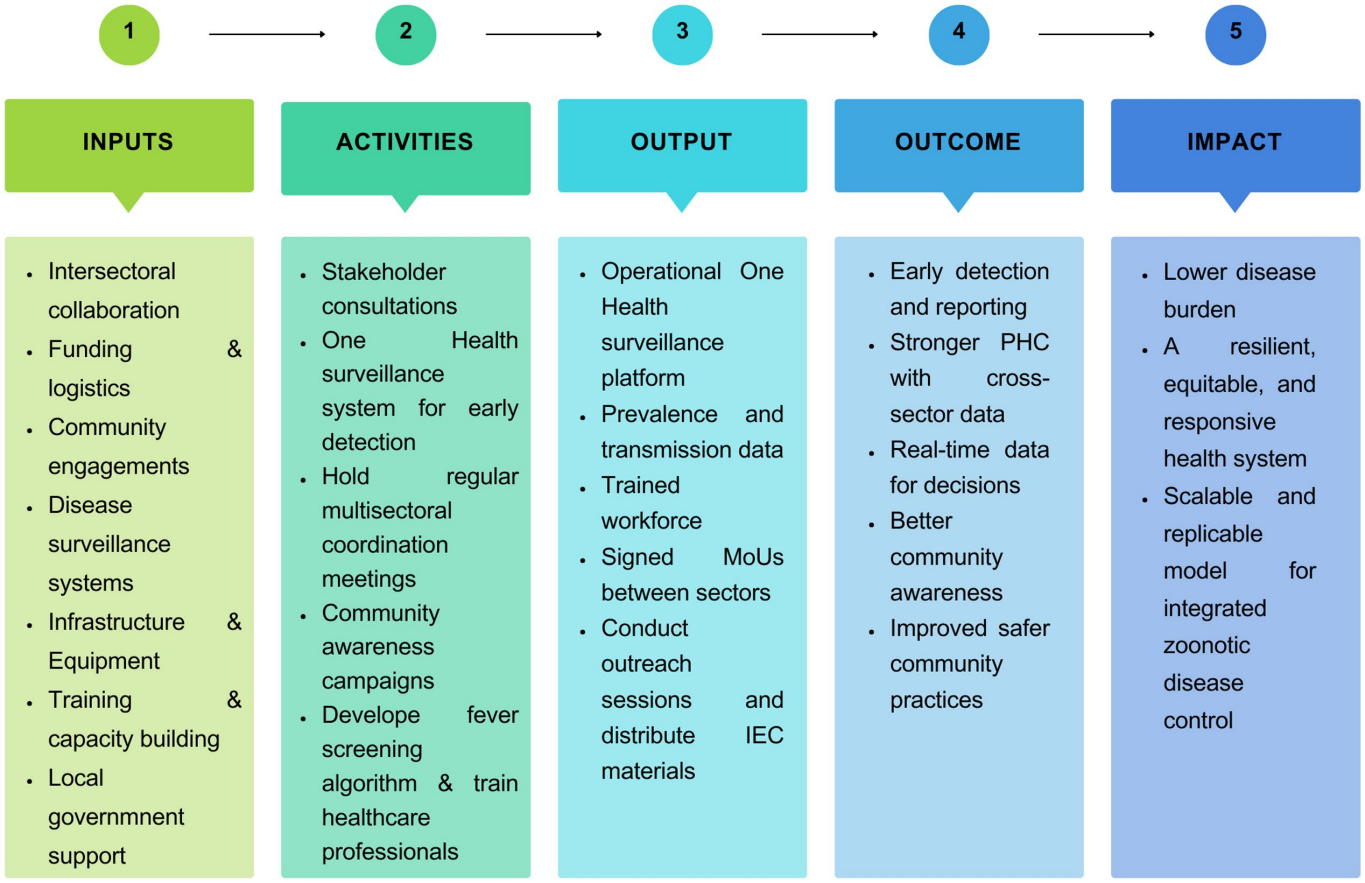
Theory of change logic model: a visual summary of inputs, activities, outputs, outcomes, and impacts within the One Health framework for controlling zoonotic diseases.

This study will use the principles of the Theory of Change (ToC) to build and test the complex intervention model for the objectives (Figure 4). Theory of change involves a thorough illustration and description of how and why desired changes are expected to happen in a specific context. It helps to connect the dots between what a programme does and the goals it wants to achieve. The construction of a ToC typically occurs through a consultative process, requiring stakeholders to reflect on how their programmes can bring about change. The following interventions will be developed to achieve the goal:

- Collaborations among departments of health, animal husbandry, environment/forest and the community.
- Assessment of the sustainability of various financed national health plans and priorities in peri-urban settings.
- Improved PHC through enhanced uptake of innovations and availability and use of timely and reliable health data for decision-making.
- Strengthen surveillance and tracking systems for early reporting of zoonotic diseases by developing and implementing a One Health-based community surveillance system.

#### 2.11.1 Public involvement

Community awareness programs and meetings with different stakeholders and community gatekeepers, like GP, self-help groups, and local leaders, will be held to bring together the community and health workers to discuss the findings and encourage community participation. The public and patients will not be involved in the study’s conception or implementation. After the completion of the study, findings will be disseminated to the participants through community-level meetings, where attendees will be encouraged to provide feedback to inform future programme planning and implementation.

#### 2.11.2 Awareness programs in villages

Repeated awareness programs regarding zoonotic diseases will be conducted in all six villages with the involvement of all the stakeholders listed in Table 2. Pamphlets will be developed in local languages and distributed in the villages to sensitise the villagers about brucellosis, leptospirosis and scrub typhus. Posters and miking vehicles will be deployed to circulate the utility of such a cohort and minimize dropouts. Pre- and post-intervention surveys, along with feedback sessions, will be conducted to assess any improvement in knowledge, attitudes, and practices of the community members.

**Table 2:** Identified stakeholders from the human and animal health sectors, administrative bodies, and the community to be included in this integrated intervention against targeted zoonotic diseases.

Health care sector	Animal care sector	Community
CDM&PHO, DPHO, DPM, DDM	CDVO	Block development administrative body
Block medical officers, Sector MO, BPM, PHEO	BVO	Chairman
CHO, ANM, ASHA Supervisor	Livestock Inspectors	Sarphanch/Naib Sarpanch
Multipurpose health worker (male & female)	Prani Mitra	Ward member, Anganwadi worker
ASHA worker	Private Practitioners	Village residents

CDM&PHO: Chief District Medical and Public Health Officer; DPHO: District Public Health Officer; DPM: District Program Manager; DDM: District Data Manager; CDVO: Chief District Veterinary Officer; MO: Medical Officer; BPM: Block Program Manager; PHEO: Public Health Education Officer; BVO: Block Veterinary Officer; CHO: Community Health Officer; ANM: Auxiliary Nurse Midwife; ASHA: Accredited Social Health Activist.

#### 2.11.3 Community discussions

Four focus group discussions (FGDs), each comprising 6–8 participants and ten in-depth interviews (IDIs) will be conducted with health care professionals and community people in study villages. Participants for FGDs and IDIs will be selected using maximum variation sampling to ensure diverse perspectives across different age groups, caste, religion, and professional backgrounds. Data collection will continue until thematic saturation. After each round of data collection, data will be thoroughly reviewed by two independent team members, and saturation will be formally recorded when no new themes are identified. A detailed list of the participants is provided in Table 3. The broad themes of the FGDs and IDIs will be based on the following two objectives.

- To assess the community’s literacy and behaviour related to WASH practices.
- To identify knowledge gaps, cultural beliefs, and key factors influencing public health behaviour related to zoonotic diseases prevention and control.

**Table 3:** Participants of community engagement (mitigating the gap due to societal intersectionality), who will be approached to assess the barriers and facilitators for co-creating the integrated intervention.

In-depth interviews	
Kakarudrapur	1. Upper caste, Hindu, woman, adult (less than 50 years) 2. Schedule caste Hindu, woman, adult (more than 50 years) 3. Veterinary doctor (private)
Jhintisasan	1. PHC doctor 2. Upper caste Hindu, woman, adult (less than 50 years) 3. Muslim, male, adult
Sathilo	Government veterinary doctor
Gandilo	Private MBBS Doctor
Alisasan	Scheduled caste, Hindu, male, adult (less than 50 years)
Chadaebar	PHN
Kakarudrapur	1. Upper caste, Hindu, woman, adult (less than 50 years) 2. Schedule caste Hindu, woman, adult (more than 50 years) 3. Private veterinary doctor
Focus group discussions	
Kakarudrapur	ASHAs
Gandilo	ANMs
Alisasan	Scheduled caste, Hindu, male adults (more than 50 years)
Sathilo	Upper caste Hindu women, adult (less than 50 years)

PHC, Primary Health Centre; PHN, Public Health Nurse; ASHA, Accredited Social Health Activist; ANM, Auxiliary Nurse Midwife.

### 2.12 Data collection and management

Open data kit (ODK ver. 2023.3.6) will be used to make online data entries and to link data from all the waves. For sample tracing from the field, as well as to maintain the flow of samples within the laboratory, unique identities and bar codes will be employed. Electronic instruments like tablets and voice recorders will be used for data entry in both qualitative and quantitative studies. Survey staff will enter the lab results into the electronic database. All survey data will be stored on password-protected tablets or computers, with access limited to authorized staff. Only the data monitoring members and the principal investigator will have access to the entered data. FGDs and IDIs will be translated and transcribed to facilitate analysis using NVivo (v14). To ensure intercoder reliability, two independent researchers will code the transcripts, develop an initial codebook, refine it through comparisons and resolve the discrepancies through discussions. Various themes and sub-themes will be generated from participants’ narratives using the inductive approach, without imposing existing theories, and will be analysed by the thematic framework. Audio recordings, transcripts, and translations will be stored securely, and encrypted backups will be maintained to prevent data loss.

### 2.13 Development of the application and integrated surveillance system

We shall establish a surveillance system for the three target zoonotic diseases, in collaboration with human and veterinary health systems. For this initiative, a fever screening tool will be developed and piloted in real-time in the first year of the study, utilising the ODK toolkit. Issues associated with the tool will be iterated based on observations from the field visits. At the end of the second year, a digital application named JEEVOne app (Joint Effort for Epidemiological Vigilance for One Health) will be developed based on a fever-tracking algorithm in collaboration with ICMR, Dibrugarh, and NESAC, Shillong, which will be provided to primary-level health workers.

In the final year of the study digital application will be subjected to a formal piloting process in selected sites to measure its usability, operational feasibility, and general acceptability. Field testing will involve close observation of real-time usage, user interviews, and monitoring of system performance under different conditions, such as where connectivity is limited. Before rolling out the app in all sites, recommendations from the users will be taken regarding the app interface and operational challenges. In addition to this, the content validity of the application will be assessed through expert reviews to ensure that the content (symptoms and exposures) included in the app to capture the disease is relevant and evidence-based. In accordance with the Digital Personal Data Protection (DPDP) Act of 2023 in India, the app will be developed with stringent user privacy protections and data security procedures, ensuring that all health and personal data is anonymized, encrypted, and accessible only by authorized staff. The app will be integrated with existing human and veterinary workflows to ensure that data collection, reporting, and follow-up align with the system, thereby facilitating adoption and sustainability.

### 2.14 Data processing

Using suitable descriptive and summary measures, quantitative and qualitative data will be evaluated and aggregated to describe the One Health concept. We will use STATA (StataCorp LLC, TX, USA) and an open-source geographic information system (GIS) software QGIS for quantitative data analysis, plotting the incidence cases and establishing causal relations between the incidence and the environment. According to the degree and pattern of missingness, missing data will either be dropped or imputed using statistical techniques such as multiple imputation or complete case analysis. Individuals who did not take part in the entire study will not be included in the analysis. Attrition rates will be calculated to assess differential dropout, and baseline traits of completers and non-completers will be compared. Depending on the variable type, comparisons will be made using chi-square tests/t-tests. Additionally, we will investigate the variations in outcome variables using relevant statistical techniques, such as regression models and time series analysis. Finally, all quantitative data will be combined to create a ‘One Health’ model in which relationships between illness patterns, health-seeking behaviour, prescribing, and resistance from various sources – human, animal, and water will be linked and tracked over time. Univariate descriptive statistics and odds ratios will be calculated. Potential confounders will be handled by including them in the multivariable models after testing for collinearity. Multivariable, stepwise conditional logistic regression will be performed to determine independent risk factors for each disease condition. In addition to this, we will employ Generalised Estimating Equations to assess the population average relationship between exposures and disease outcomes.

Integrating quantitative and qualitative data by specific mechanisms such as data transformation, joint display, convergent design, sequential exploratory design, and data triangulation would enhance the robustness of the study. A comprehensive strategy will be developed for disseminating study findings to relevant stakeholders on a local and global scale, taking into account diverse communication channels such as peer-reviewed publications, conference presentations and involvement with mainstream and social media. In addition, focused outreach efforts will be made to policymakers, healthcare professionals, and community groups to ensure that the findings are accurate. The study will be reported according to the STROBE (Strengthening the Reporting of Observational Studies in Epidemiology) guidelines to ensure transparency and completeness of the data.

### 2.15 Ethical considerations

The study protocol has been approved by the Institutional Ethics Committee of the ICMR-RMRC, Bhubaneswar (Ref No: ICMR-RMRC/IHEC-2022/128) and will follow the National Ethical Guidelines for Biomedical and Health Research Involving Human Participants by the ICMR, New Delhi. The State Ethics and Review Committee, Department of Health and Family Welfare, Government of Odisha also approved this project proposal (Ref No. 25588 dated 25/10/2023). We will ensure prior permission from the local administration for fieldwork. All participants will be briefed on the study’s objective, and a written ICF will be obtained before the interview. The participation was purely voluntary, and the respondent could withdraw from the interview at any point in time without citing any reason. Written informed consent will be obtained from participants before they participate in the study, and they will be provided with a participant information sheet (PIS) for their information. For domestic animals, written informed consent will be obtained from the owners. To ensure confidentiality, personal identifiers will be removed during data entry and analysis.

## 3 Anticipated results

### 3.1 Result 1: assessment of zoonoses epidemiology in spatial and temporal dimensions

The serological data from both humans and animals will elucidate the prevalence, distribution pattern and transmission dynamics of brucellosis, leptospirosis, and scrub typhus. It will also identify the associated risk factors based on socio-economic conditions, perceptions, and knowledge of zoonoses, availability of essential amenities, daily lifestyle, and other environmental factors. The spatial data will help in mapping zoonotic disease hotspots, identifying risk zones, and predicting disease emergence through integrating multisectoral data. This One Health approach will lead to enhanced stewardship, compliance, and interspecies equity (balanced health benefits across species), enabling humans to benefit in the long term from their domestic animals and ecosystems. The conceptual framework will predict disease onset more efficiently, with improved community awareness and robust communication among responsible stakeholders that facilitate rapid action on illness. This epidemiological data will aid in the development of evidence-based policy through the processes of identifying vulnerable populations, policy experimentation, policy evaluation, implementation optimisation, and long-term impact assessment.

### 3.2 Result 2: optimization of digital application to track the cases

The primary purpose of using this application will be to facilitate the early screening of individuals with probable and specific symptoms of brucellosis, leptospirosis, and scrub typhus. Once someone is identified as a probable case, both the human and veterinary health systems will be informed to take immediate action for diagnosis. It will also include questions that help identify the source of infection, especially if it originated from an animal. Additionally, it will help to determine how many people may have been exposed to the infected source. This digital application-based integrated surveillance system will assist in:

1. Outbreak preparedness
  - Notifying and keeping track of an outbreak progression
  - Source identification and neutralization
  - Mapping of the outbreak and associated risk factors
2. Early screening and mobilisation of the suspected cases
3. Follow up on the new cases

### 3.3 Result 3: translational value of the intervention packages with decision output

The study outcome will enhance the capacity of community members and key stakeholders by convening and consulting experts from diverse fields to effectively assess a peri-urban cohort for addressing zoonotic diseases with a One Health approach. The findings will directly inform the formulation and adaptation of intervention strategies like animal vaccination, WASH improvements or behavioural changes. The intervention packages, based on the ToC approach, will be tailored to local contexts and will address specific environmental, veterinary, and behaviour-related issues. The active participation of various experts: clinicians, veterinarians, and public health professionals will help ensure that the interventions are evidence-based, culturally appropriate, and feasible to implement. The policy decisions are expected to be useful for district- and state-level governance by presenting viable models for interdisciplinary disease surveillance and prevention. By anchoring the interventions in locally relevant socio-ecological realities while aligning them with national health priorities, the study aims to bridge existing gaps between research, practice, and governance.

## 4 Discussion

This study aims to integrate public health, veterinary, and environmental sectors by developing a One Health surveillance system to monitor zoonotic pathogens. Through a prospective cohort approach, the study will generate data on the prevalence, transmission dynamics, and risk factors associated with scrub typhus, leptospirosis, and brucellosis in peri-urban Odisha. This multi-prospective cohort study will follow a conventional approach of tracking individuals and animals from the community and their environment to provide longitudinal insights into disease patterns. It will generate surveillance data to guide prevention and control efforts for these zoonotic diseases. Understanding pathogen ecology, ecosystem changes, high-risk human behaviours, real-time data sharing and reporting, One Health integration, and socioeconomic risk perception will be the necessary outcomes to prevent future zoonotic spillover events. GIS mapping will be used to visualise high-risk zones, guiding targeted disease prevention strategies. The findings will contribute to evidence-based interventions for zoonotic disease control in peri-urban settings.

A key finding of this study will be a better understanding of the epidemiology of zoonotic diseases in such transitional zones, which are often overlooked by public health surveillance. Further, this study will examine the role of WASH in zoonotic disease transmission and assess its impact on zoonotic disease spread and identify modifiable risk factors. Qualitative and quantitative assessments will help identify key transmission pathways, particularly among livestock owners and handlers. This study will also identify potential environmental reservoirs of zoonotic pathogens and their implications on human and animal health. Expected findings may indicate whether close animal contact, water contamination, or occupational exposure are primary risk factors.

This study has some limitations as well. Rapid urbanization and change in land use patterns are characteristic features of peri-urban settings, which may impact the disease transmission dynamics over time. Engagement of the community and compliance with the surveillance activities will require continuous stakeholder involvement and may pose challenges in the behavioural change interventions. Integration of multi-sectoral data from human, animal and environmental sources will require robust analytical frameworks and interdepartmental coordination.

This study will serve as a foundation for a multi-centric One Health surveillance model, with the potential for replication in other regions of India. It also aligns with national disease surveillance programmes, such as the Integrated Disease Surveillance Project (IDSP), in the management of various zoonotic diseases. Understanding the role of emerging zoonotic pathogens in disease dynamics, along with evaluating community-based and disease-specific interventions, will open new avenues for future research. This study will contribute to a holistic and evidence-based public health approach that integrates human, animal, and environmental health. The outcomes of the study will help strengthen disease surveillance systems, inform disease-specific policy decisions, and foster cross-sectoral collaboration for improved health security in peri-urban settings. This model holds potential to extend beyond peri-urban India, offering a scalable framework for other LMICs with comparable ecological and health system challenges. Its adaptability can make it a valuable tool for strengthening zoonotic disease prevention globally.

## Footnotes

1
www.osdma.org
