# Supplementary material for: Development of One Health-based community surveillance system to study zoonotic diseases: a study protocol
Source: Front Public Health. 2026 Jan 14;13:1646123. doi: 10.3389/fpubh.2025.1646123 (PMC12848608; doi:10.3389/fpubh.2025.1646123)
Supplement: Supplementary file 1 [file Supplementary_file_1.docx]

**Table S1.** Prevalence of target diseases among participants in pilot study.

| **Disease** | **IgM positive (%)** | **IgG positive (%)** |
| --- | --- | --- |
| Scrub Typhus | 7 (7.8)^*^ | 64 (71.1) |
| Brucellosis | 14 (15.6) | 35 (38.9) |
| Leptospirosis | 25 (27.8) | 23 (25.6) |

**^*^**used for sample size calculation

**Table S2.** Details of serological test kits to be used in this study. ELISA: Enzyme-linked immunosorbent assay; RBPT: Rose Bengal Plate Test; MAT: Microscopic Agglutination Test.

| **Target Disease** | **Sample type** | **Test** | **Antibody** | **Name of kit & manufacturer** | **Approx Test time (min)** |
| --- | --- | --- | --- | --- | --- |
| Brucellosis | Human serum | ELISA | IgG | Brucella IgG ELISA, Calbiotech | 150 |
|  |  |  | IgM | Brucella IgM ELISA, Calbiotech | 150 |
|  | Animal serum | ELISA | IgG | In-house kit by ICAR-NIVEDI, India | 240 |
|  |  | RBPT | IgG | In-house kit by IAHVB, Bengaluru, India | 10 |
| Leptospirosis | Human serum | ELISA | IgG | NovaLisa® Leptospira IgG, NovaTech | 180 |
|  |  |  | IgM | Panbio™ Leptospira IgM ELISA, Abbott | 195 |
|  | Animal serum | MAT | IgG | As per standard protocol by WOAH and WHO | 240 |
| Scrub typhus | Human serum | ELISA | IgG | Scrub Typhus Detect™ IgG ELISA, InBios | 180 |
|  |  |  | IgM | Scrub Typhus Detect™ IgM ELISA, InBios | 180 |
